# Emotion Recognition from Chinese Speech for Smart Affective Services Using a Combination of SVM and DBN

**DOI:** 10.3390/s17071694

**Published:** 2017-07-24

**Authors:** Lianzhang Zhu, Leiming Chen, Dehai Zhao, Jiehan Zhou, Weishan Zhang

**Affiliations:** 1 Department of Software Engineering, China University of Petroleum, No. 66 Changjiang West Road, Qingdao 266031, China; chenleiming1930@sina.com (L.C.); zhaodh.upc@gmail.com (D.Z.); 2 Department of Information Processing Science, University of Oulu, Oulu FI-91004, Finland; jiehan.zhou@oulu.fi

**Keywords:** speech emotion recognition, speech features, support vector machine, Deep Belief Networks

## Abstract

Accurate emotion recognition from speech is important for applications like smart health care, smart entertainment, and other smart services. High accuracy emotion recognition from Chinese speech is challenging due to the complexities of the Chinese language. In this paper, we explore how to improve the accuracy of speech emotion recognition, including speech signal feature extraction and emotion classification methods. Five types of features are extracted from a speech sample: mel frequency cepstrum coefficient (MFCC), pitch, formant, short-term zero-crossing rate and short-term energy. By comparing statistical features with deep features extracted by a Deep Belief Network (DBN), we attempt to find the best features to identify the emotion status for speech. We propose a novel classification method that combines DBN and SVM (support vector machine) instead of using only one of them. In addition, a conjugate gradient method is applied to train DBN in order to speed up the training process. Gender-dependent experiments are conducted using an emotional speech database created by the Chinese Academy of Sciences. The results show that DBN features can reflect emotion status better than artificial features, and our new classification approach achieves an accuracy of 95.8%, which is higher than using either DBN or SVM separately. Results also show that DBN can work very well for small training databases if it is properly designed.

## 1. Introduction

Emotion is a mixture of people’s physiological response and inner thoughts and plays an important role in rational actions and decision making for human beings. Automatic emotion recognition from speech has been an active research topic for applications such as smart health care, smart home, smart entertainment, and many other smart services. For example, monitoring of emotion in a call center reminds staff to adjust their service manner when someone has a negative mood. However, emotion recognition is a complex task because it is hard to identify what the correct emotion is for a given sample, and the distinction between different emotional tones is very narrow.

Much research has been done towards recognizing human emotions using speech information [1,2,3]. However, there is little research on emotion recognition in Chinese speech, which is more challenging because of complexities arising from Chinese language’s prosodic characteristics. The work in [4] tried to find robust speech features for emotion recognition in Chinese speech. However, they analyzed only intrinsic speech features, not statistical features. Intrinsic speech features represent the attribute of a speech sample, and statistical features reflect relationships between them. Statistical features such as maximum, minimum, mean, variance and standard deviation should be taken into consideration in speech emotion recognition.

There are two key points that impact the performance of speech emotion recognition [4,5,6,7,8]:

- The first is speech feature selection.Because there are many kinds of features that can be extracted from a speech sample, it is difficult to know which one should be chosen as the most suitable for emotion recognition. Some work [1,2,4,5,9,10,11] shows that prosody features (i.e., pitch, energy, Zero crossing rate) are important, other work [4,5,8,9,10] shows that quality features (i.e., Formant Frequencies, Spectral features, etc.) are helpful for speech emotion recognition. Researchers have also found that derived features (i.e., Mel-Frequency Cepstral Coefficient (MFCC), Linear Predictive Coding Coefficients (LPCC)) can be critical [1,5,6,12,13], and that dynamic features such as Mel-Energy spectrum dynamic Coefficients (MEDC) contribute to speech emotion recognition [5]. Existing research usually combines these features as vectors to reflect speech samples’ emotion status. But this approach has limitations. The first issue is how to best combine these features, as different features may have different contributions. The second issue is that these features may not be the most suitable features. The last issue is that feature extraction algorithm’s parameters are adjusted “manually”, which increases the influence of subjective factors.
- The second point affecting emotion recognition accuracy is what kind of classification method is used to do the recognition. Classification methods include: SVM based approach [4,5,7,13,14,15], GMM based approach [9], ANN approach [6,16], RNN [1] and BayesNet based approach [4,15]. Although some deep models such as RNN have been applied, most of work in this filed use shallow classifiers, which can not detect the deep features in speech signals. Deep features are features extracted by deep neural networks and hence the name, which contains more information than artificial features such as MFCC and LPCC.

Deep Belief Network, as one of the important deep learning techniques, is a probability generation model that can be used to extract deep features from speech and image signals. Research [17] has shown that DBN can take the place of a GMM universal background model to derive a vector representation of an utterance which is then used for vector-based classification. SVM is known as one of the best shallow classifiers, and performs well in vector-based classification tasks. Due to the complexity of audio speech signals, traditional statistical models are limited in classifying emotional status and intelligent models such as SVM would be more effective in such recognition. So it is natural to combine advantages from both DBN and SVM, as shown in the research in [18], which demonstrated that SVM can achieve good results when solving feature vectors extracted by deep neural networks.

However, previous work [19] only used SVM and DBN to conduct emotion recognition and compared their performance, so this work tried to take advantage of these two algorithms to form a new method, which is a combination of DBN and SVM for accurate emotion recognition from Chinese speech. We extracted five of the most widely used speech features (pitch, short-term energy, short-term zero-crossing rate, formant and MFCC), then compared the contribution of intrinsic features with statistical features, and tested if it helps to improve classification accuracy when using SVM and DBN. Considering the usability and validity of various classification methods, we took advantage of SVM and DBN by combining them as an end-to-end classifier. We also explored how to optimize the proposed algorithm such as hyper parameter optimization in this paper. Furthermore, a conjugate gradient method was introduced into DBN in order to speed up the training process, saving much time during parameter optimization process for DBN.

The rest of this paper is organized as follows. Section 2 gives a brief description of speech emotion recognition including speech features and classifiers. Section 3 proposes a novel classifier combining SVM and DBN. Section 4 presents evaluations of various features, especially evaluation of the proposed classification method. The paper ends with our conclusion and suggested future work.

## 2. Background of Speech Emotion Recognition

In this section, we first discuss speech features that can be used to classify emotions of speakers. Then we present two typical classification approaches that show strengths compared to other approaches, i.e., SVM and DBN.

### 2.1. Speech Emotion Features

**Figure 1:**
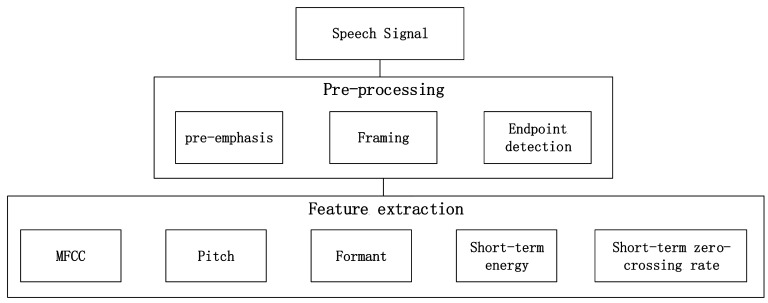
Process of extracting speech features.

Speech signals contain large amounts of information. Two of the most important types of information are verbal content and emotion status, both of which can be distinguished relatively more easily by humans than by computers. Emotional status are represented by many features, so extracting suitable features from speech signals that can effectively characterize different emotions is crucial. Generally, five kinds of features are considered as the most useful features to reflect a speaker’s emotion: pitch, short-term energy, shot-term zero-crossing rate format and Mel frequency cepstrum coefficient. The feature extraction process is shown in Figure 1. The speech signal should go through a pre-processing step which aims to remove the noise and other irrelevant components of speech corpus for better input data. The pre-processing step consists of three major parts such as pre-emphasis, framing and endpoint detection. In the pre-emphasis step, a Finite Impulse Response (FIR) filter which is also called pre-emphasis filter is used on the speech signal. The impulse response of the filter is given by:

(1) $$H\left(z\right)=1+az^{-1},a=-0.9375$$

After filtering through the pre-emphasis filter, the speech signal is divided into windows of 25 ms with an overlap of 10 ms, and each divided part is taken as a frame. Then each frame is applied a hamming window for the reduction in discontinuity of the speech signal, which can also avoid leakage in a frequency spectrum. Endpoint detection can determine the end of each speech signal.

**Figure 2:**
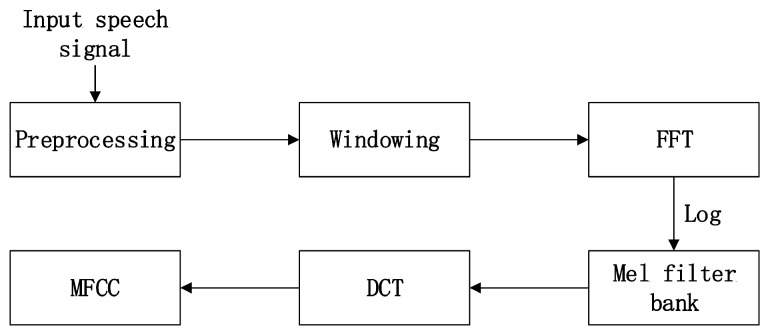
Process of extracting Mel-Frequency Cepstral Coefficient (MFCC).

Each extracted feature has its unique contribution to speech emotion recognition and the extraction methods are described as follows:

- Pitch frequency
  Pitch frequency is caused by speaker’s vocal chords vibrating. It is one of the most important features to identify excitation in speech signal analysis. There are various methods such as the autocorrelation method, average magnitude difference function, wavelet transform and the cepstrum method to extract pitch. We choose the cepstrum method because it can avoid the interference of formant. The cepstrum of signal $s\left(n\right)$ can be defined as the inverse discrete Fourier transform of its frequency spectrum’s natural logarithm:
  (2) $$\hat{s}\left(n\right)=IDFT\left\{\ln\left|DFT\left[s\left(n\right)\right]\right|\right\}$$
- Short-term energy
  Short-term energy reflects the amplitude characteristic of speech signal, which can be used to distinguish between voice and noise. The *n*-th frame’s short-term energy $E_{n}$ is calculated as follows, in which $x_{n}\left(m\right)$ represents the m-th data point in the *n*-th frame:
  (3) $$E_{n}=\sum_{m=0}^{N-1}\left|x_{n}\left(m\right)\right|$$
- Short-term zero-crossing rate
  Short-term zero-crossing rate (ZCR) indicates the number of times a speech signal wave crosses the horizontal axis in each frame, which can be used for endpoint detection and mute elimination. The *n*-th ZCR can be calculated by observing the positive and negative change of speech signal, in which sgn[] is a symbolic function.
  (4) $$ZCR_{n}=\frac{1}{2}\sum_{m=0}^{N-1}\left|sgn\left[x_{n}\left(m\right)\right]-sgn\left[x_{n}\left(m-1\right)\right]\right|$$
- Formant
  Generally, formant is extracted according to the position and bandwidth attribute of the speech signal, which is usually applied to distinguish vowels and changes with different pronunciation mechanisms. A linear prediction method is accepted as being the most effective in various formant extraction algorithms. Using this method, future samples can be extracted by observing the previous *k* samples. The loss function $e\left(n\right)$ is shown below, in which $a_{i}$ is the model parameter, and the goal is to adjust $a_{i}$ to minimize the loss function:
  (5) $$e\left(n\right)=s\left(n\right)-\sum_{i=1}^{k}a_{i}s\left(n-i\right)$$
- Mel-frequency cepstrum coefficient (MFCC)
  MFCC is one of the most widely used features for speech analysis. It is extracted based on the human auditory system, which provides a natural and real reference for speech recognition. The calculation process of MFCC is shown in Figure 2. After preprocessing, the speech frame passes through a hamming window, then the energy distribution is calculated by a fast Fourier transformation. A Mel filter bank is used to eliminate the effect of harmonics. The last step is discrete cosine transformation. An *N* dimension MFCC feature consists of four parts: basic MFCC, the first difference parameters, the second difference parameters and energy.

Although many speech features can be extracted, they are still single-frame characteristics which are independent of their relationships to the whole speech sequence. In fact, these relational characteristics may be critical to identify the emotional content of speech. Therefore, for each speech feature we propose to measure its relational characteristics by calculating five statistical features (maximum, minimum, mean, variance and standard deviation) , and then analyze each of them to identify which one is the most important feature.

### 2.2. Approaches to Classification

SVM is a dichotomous model [20], originated in statistical learning theory, which offers robust classification of a very large number of variables even with small samples [21]. In addition, it can learn complex data from classification models applying mathematical principles to avoid overfitting [15]. SVM has been used widely for classification because of its advantages of simplicity and capability.

#### 2.2.1. Principles of SVM Multi-Classification Algorithm

An SVM kernel function maps linearly non-separable data to a high dimension feature space and aims to find the hyperplane to make different samples have the maximum distance. When facing a multi-classification task, there are mainly two schemes:

- A multi-classification problem is divided into a series of dichotomous problems which are easier to solve.
- The original dichotomous SVM model is modified so that it can solve the multi-classification problem.

Although the second method can solve a multi-classification problem directly, the parameter optimization is more complicated than the first method and the calculation is very large. Therefore the first approach is usually used in practical applications, which typically has one of three forms: one versus rest, one versus one and decision tree.

During the training process, there are two key parameters: penalty factor sig2 and kernel parameter gam, which are related to dataset size and training iteration. A large penalty factor can lessen error but will lead to overfitting. On the contrary, a small penalty factor will lead to underfitting. These parameters are usually optimized by cross-validation on training datasets. Kernel parameter gam is used in Radial Basis Function (RBF) kernel, which is also taken as a Gaussian kernel function, shown as follows.

(6) $$k\left(x,z\right)=exp\left(-gamma\cdot d{\left(x,z\right)}^{2}\right)$$

In this kernel function, the relationship between sigma and gamma is:

(7) $$gamma=1/2\cdot \sigma^{2}$$

Generally, a Gaussian function is applied when solving high-dimension data, such as the features extracted from speech in this research. However, a Linear kernel function can not classify high-dimension data, which is known as linearly non-separable problems. A Gaussian function with a small σ is tall and thin in shape, leading to a small range of support vector sample and poor performance of classifying unknown samples. However, the smoothing effect will be serious when using a too small σ, causing low accuracy on both training dataset and test dataset.

The SVM based classification followings four steps as below:

- Initialize sig2 and gam to 2 by experience.
- Train SVM using the input dataset and change the value of sig2 and gam.
- Analyze the output and record the corresponding sig2 and gam value with the highest accuracy.
- Update parameters of SVM with the recorded sig2 and gam value and repeat step 2 until achieving the best accuracy result.

#### 2.2.2. Deep Belief Network (DBN)

DBNs are being widely used for classification and feature extraction [22]. A DBN consists of multi-layer Restricted Boltzmann Machine (RBM). RBM is a bipartite graph with Boltzmann probability distribution, which contains a visible layer and a hidden layer. These two layers are connected by weights but nodes in the same layer are unconnected. DBN is trained by back propagation (BP), which is similar to other multi-layer neural networks.

**Figure 3:**
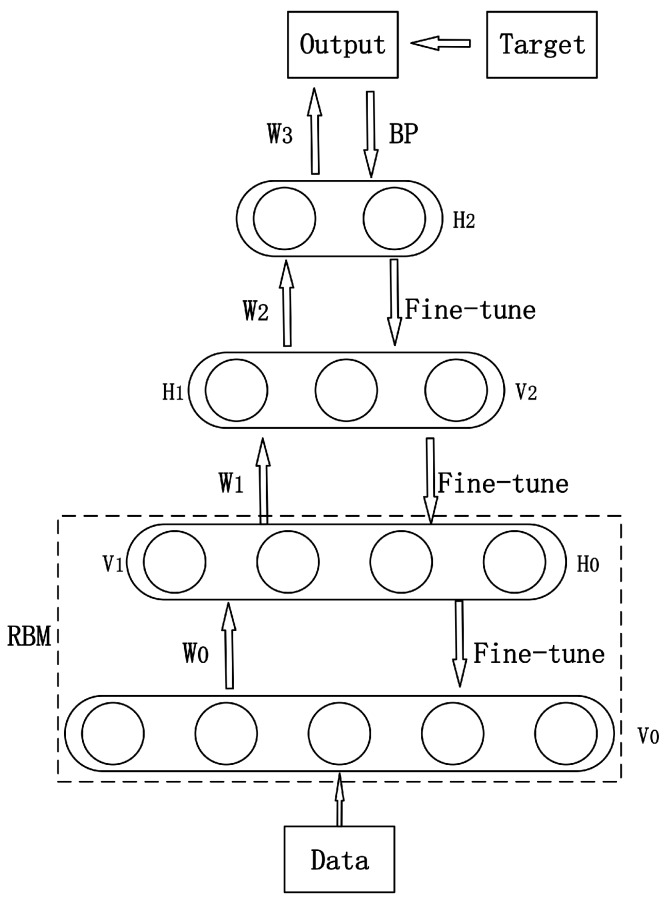
Structure of Deep Belief Network (DBN).

A typical DBN model is shown in Figure 3. In the first phase, the input data is used to conduct forward-propagation on RBM in each layer. When feature vector mapping to different feature space, information is retained as much as possible so that it can be converted into high level abstraction. $V_{0}$ is the first visible layer and the input layer as well. $W_{0}$ is the parameter learned from training data, using which the hidden layer $H_{0}$ is rebuilt, and it also acts as the second visible layer $V_{1}$. In the second phase, supervised learning with a BP algorithm is used to train softmax in the top layer and fine-tune the parameters in the network.

DBN has been successfully used for modeling the posterior probability of a given feature vector (e.g., MFCC). The parameters of DBN are given by *W* (connection weights), *b* (visible-unit bias) and *c* (hidden-unit bias). And the probability of input vector *v* and output vector *h* is given by:

(8) $$p\left(v,h\right)=\frac{e^{-E\left(v,h\right)}}{Z}$$

where $E\left(v,h\right)$ is the energy function:

(9) $$E\left(v,h\right)=-b^{T}v-c^{T}h-h^{T}Wv$$

and *Z* is normalizing factor obtained by summing the numerator of (8) over all possible status of *h* and *v*.

(10) $$Z=\sum_{v,h}e^{-E\left(v,h\right)}$$

Avoiding complex work of feature extraction and selection, a DBN can effectively generate discriminative features that approximate the complex non-linear dependencies between features in the speech samples. Audio features in the input layer are learned in the hidden layers, and the learned features act as the input of the next layer. The output layer finally gives a class for the input sample.

## 3. Combining DBN and SVM for Speech Emotion Recognition

As discussed in the introduction, previous work has shown that feature extraction and classification methods selection are two main factors which influence performance of speech emotion recognition. Existing approaches usually use artificial features but they may loss some important information. The parameters of a feature extraction algorithm are adjusted by experience and the process is complicated. In addition, these speech features are shallow and would be impacted easily by noise. In addition, different classification methods have unique advantages and disadvantages. Usually shallow models are simple to be used and there are few parameters need to be adjusted, but they require good feature representations to distinguish different emotional status. Deep neural networks (e.g., DBN) can automatically learn deep features from raw speech data, saving much manual work of extracting various speech features.

**Figure 4:**
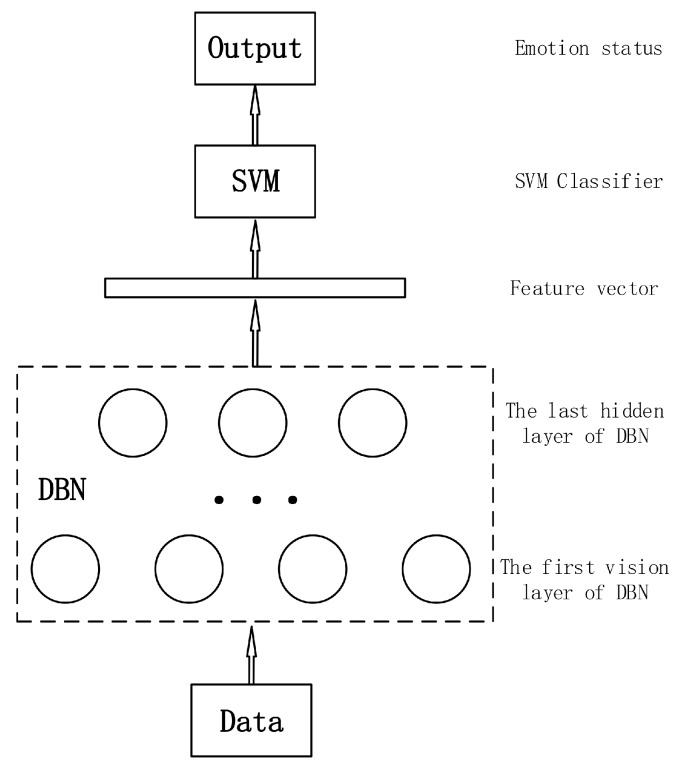
Structure of combining support vector machine (SVM) and DBN. Speech features are converted into deep features by a pre-trained DBN, which are feature vectors output by the last hidden layer of the DBN. The feature vectors act as the input of SVM and are used to train the SVM. The output of the SVM classifier is the emotion status corresponding to the input speech sample.

In order to achieve an effective and efficient speech emotion recognition, we propose a novel classification approach that takes advantage of DBN and SVM. Figure 4 shows the structure of the proposed approach. Firstly, DBN is used to extract deep speech features from raw speech samples. However, the top layer is replaced by SVM. Then SVM is trained using deep speech features which are actually feature vectors. That is to say, the output of DBN acts as the input of SVM and they are merged as an end-to-end classifier. The proposed classifier possesses common advantages of both SVM and DBN, and can achieve ideal results without much work on parameter optimization.

Generally, a good network model could be achieved by adjusting parameters over and over again. However, With the increase of dataset size, long training time becomes a crucial problem. Therefore a conjugate gradient method is introduced into the training process in order to speed up training process which aims to minimize Mean Square Error (MSE) between output *o* and label $o_{d}$ by calculating the optimal solution of the weight matrix.

(11) $$MSE=E\left(e^{T}e\right)=E\left[{\left(o_{d}-o\right)}^{T}\left(o_{d}-o\right)\right]$$

At the beginning of training process, the gradient is initialized as:

(12) $$g_{0}=▽MSE\left(w_{0}\right)$$

Then the weight matrix is adjusted as follows, in which $d_{k}$ and $\alpha_{k}$ represents the *k*-th direction and step size respectively.

(13) $$w_{k+1}=w_{k}+\alpha_{k}d_{k}$$

The (k+1)-th direction is:

(14) $$d_{k+1}=\beta_{k}d_{k}-g_{k+1}$$

It is necessary that $d_{k+1}$ and $d_{k}$ have to be conjugated. Moreover, parameter $\beta_{k}$ is calculated as:

(15) $$\beta_{k}=\frac{{∥g_{k+1}∥}^{2}}{{∥g_{k}∥}^{2}}$$

## 4. Evaluation

### 4.1. Database

**Figure 5:**
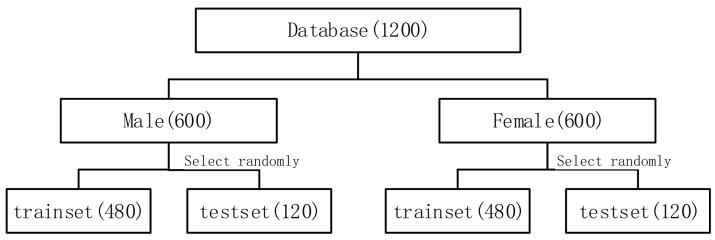
Dataset structure.

There are some widely used databases, e.g., the Berlin speech emotion database [23], and USC-IEMOCAP database [24]. We used the Chinese Academy of Sciences emotional speech database (http://www.datatang.com/data/39277) to evaluate the proposed approach. As shown in Table 1, speech samples in the database were recorded by four professional announcers, including two males and two females. These announcers have been trained specially and had rich experience in simulating different emotions. In the recording process, four announcers spoke sentences in six different emotional statuses. The total 1200 sentences contain six kinds of emotional status: angry, fearful, happy, neutral, sad and surprise and each emotional status has 50 utterances. The dataset structure were organized as shown in Figure 5. The speech data was recorded in a pure acoustic environment with 35 db signal-to-noise ratio (SNR), 16000 HZ sampling rate, 16 bit rate, and stored as PCM format. Pitch, formant, short-term energy, short-term zero-crossing rate and MFCC were extracted from the speech samples and the corresponding statistical features such as minimum, maximum, mean, variance and standard deviation are calculated. These features were used to organize different feature vectors in the following experiments.

### 4.2. Experiments with SVM and DBN

Male and female speech have many different inherent characteristics. For example, the formant features have large discrepancies between different genders, which has a significant impact on emotion recognition [25]. It is important to consider the differences between males and females, whose vocal characteristics are so disparate even when they are in the same emotional status and so would greatly affect emotion analysis, we divided the dataset into a male group and a female group and conducted emotion recognition on them separately to achieve better results. For each group, training set was used to train SVM and test set was used to test the trained model. The statistical features of each extracted speech feature were used in the experiment. They are feature vectors with the length of 14, including the maximum, minimum, mean and variance of pitch, formant and MFCC, attached with short-term energy and short-term zero-crossing rate. Statistical features are good representations of speech emotion characteristics. So they are used to train SVM.

In the test phase, we used the same number (40) of speech samples to evaluate the performance of the trained model for each emotion. So the experiment could reveal whether the model performs better for some emotions or equally for all six emotions. The 10-fold cross-validation results of the multi-class SVM classifier are shown in Table 2. It can be seen that the mean accuracy of the male and female group is 85% and 84.8% respectively, which shows that the statistical features have similar contribution to male and female speech emotion recognition.

**Figure 6:**
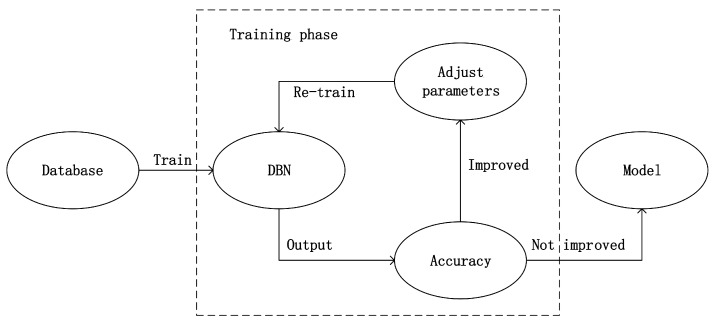
DBN training phase.

For the next experiment, we used DBN to implement emotion recognition. The dataset was also divided into two groups, the male group and the female group. However, this time speech features which is a 60 dimension vector were taken as the input of DBN. Before training, we adjusted the network structure parameters such as number of hidden nodes, number of hidden layers in order to build the best network structure. Then we adjusted the hyper parameters such as learning rate, batch size, momentum and number of iterations by trying and observing when the highest accuracy was achieved. Moreover, the network parameters were adjusted by training with back propagation (BP). As shown in Figure 6, during the training phase, the loop would stop until the accuracy could not be improved, otherwise the hyper parameters would be adjusted and DBN would be re-trained.

In the experiment, each training process contains 3000 iterations and the time consumption is 180 s for one process. Although the time consumption for each training process is low, it is difficult to choose suitable parameters for a good result. One group of parameters may be fit for recognizing anger but unfit for recognizing surprise. Therefore, training DBN was time consuming because of hundreds of times hyper parameter adjustment and we should find a group of parameters which can achieve the highest mean accuracy for recognizing all six types of emotion. The 10-fold cross-validation results of DBN are shown in Table 3.

The mean accuracy of the DBN-based approach is 94.6%, which is higher than that of SVM (84.8%). That is to say, when applying speech features to analyze speech emotion, the performance of DBN is better than SVM. Compared to SVM, which uses statistical features, DBN can learn hidden information from speech signal and so achieve higher accuracy.

### 4.3. Experiments Combining SVM and DBN

Since DBN has a good capability of converting low level speech features to high level emotion representations and the parameter optimization of SVM is simple, we can take advantage of the benefits from each one, i.e., taking the output of DBN’s last hidden layer as the input of SVM. This idea suggests a novel classification method that combines SVM and DBN. In this experiment, DBN was pre-trained using speech features in a training set. Then softmax classifier in the top layer was removed so that it can be considered as a deep feature extractor and the features it extracts can be taken deep features. We used the deep features to train SVM and adjust sig2 and gam until we achieve the best result. The 10-fold cross-validation results are shown in Table 4.

Obviously, with the mean accuracy of 95.8%, the proposed classification method has higher accuracy than that of using only SVM or DBN. It also proves that DBN can successfully extract useful features to distinguish between different emotions and when applying SVM as the classifier, and the deep features have better performance than statistical features. Additionally, when applying conjugate gradient method, it takes 150 s for 3000 iterations, which means this method can speed up the training process by about 16.6% and save time of adjusting parameters and training DBN.

## 5. Discussion

Usually, training deep neural networks needs a large amount of data. The Berlin speech emotion database is one of the most widely used databases but it contains about only 800 sentences, which is smaller than the Chinese speech emotion database used in this paper. Such a small dataset leads to problems such as overfitting. Therefore, we designed a suitable DBN model which contains 3 hidden layers for the small database.

Generally, there is not a clear regulation to adjust hyper parameters for deep belief networks, so doing continuous experiments is a good way to find out them. In our work, 3000 iterations with a learning rate of 0.01 and momentum of 0.9 performs well for this database. Using the proposed method, we achieved a mean accuracy of 95.8%, better than SVM and DBN only, which demonstrates that our approach can adapt to the small dataset well.

We conducted gender-depended experiments because of the differences in speech characteristics between male and female. Each experiment achieved similar accuracy in the male group and the female group, which proved that a suitable speech features can represent emotion status well despite of the difference of inherent speech characteristics and that relevant deep features can adapt to different genders.

Although the dataset we used was created artificially, it was hard to come by because these sentences were spoken by professional announcers. Additionally, this database was certified and released by an official Chinese institution, so the database was of high quality.

### Related work

There is a lot of good research on speech emotion recognition. In order to compare them with our work, some information can be found in Table 5.

Kumar et al. [5] introduced a novel approach of using a combination of prosody features, quality features, derived features and dynamic features for robust automatic recognition of the speakers’ emotion status. They used a multilevel SVM classifier to identify seven kinds of emotions in ‘Five native Assamese Languages’ and finally achieved an accuracy of 82.26% for speaker independent cases. They used more features than we did but their features were extracted by fixed algorithms instead of being extracted automatically by the deep neural networks.

Huang et al. [9] focused on insight into emotion changes instead of analyzing a single speech file. They detected the instant of emotion change using GMM based method on the IEMOCAP database. The Generalized Likelihood Ratio and Emotion Pair Likelihood Ratios, together with a novel normalization scheme are applied to improve emotion change detection accuracy. The best Equal Error Rate is 20.2%, which is lower than ours.

In [26], Zhang et al. proposed a novel method called cooperative learning, which consisted of combining Active Learning and Semi-Supervised Learning techniques and aimed to reduce the costly effects of human annotation. Their work led to the same performance of a model trained on the whole training set, but used 75% fewer labeled instances. We used DBN to extract deep features in order to reduce the complex manual work, and we achieved a higher accuracy than them (66.7%).

Rawat et al. [6] used Neural Network as a classifier to classify the different emotion from speech data. They extracted MFCC as the input of the Neural Network. In addition, a high pass filter was applied to reduce noise and increase performance. They achieved a very high mean accuracy of 93.38% for distinguishing five kinds of emotion status. We did not use any filter to reduce noise because our database was recorded in pure acoustic environment. However, in any practical application, noise reduction is important and should be taken into consideration.

Li et al. [4] investigated the effects of the commonly utilized spectral, prosody and voice quality features in emotion recognition with three types of corpus, to find the most robust feature for emotion recognition from natural speech. They also compared different machine learning methods including SVM, J48, Random Forest, RBFnerwork and BayesNet. Their best result was achieved by SVM, which had an accuracy of 84.8%. In contrast to their work, we not only considered statistical features and deep features, but also compared the contribution of them, and we also achieved a good result using SVM only.

Lee et al. [1] took into account of the long-range context effect and the uncertainty of emotion label expression and presented a speech emotion recognition system using RNN. To overcome the uncertainty of emotional labels, they assumed that the label of each frame is regarded as a sequence of random variables. An improvement of 12% compared to the DNN-ELM based method was finally achieved. Although we used deep feature and SVM to achieve a better result, their work of considering the long-short term memory is a good reference for improving our work.

Wang et al. [3] proposed a novel feature extraction method based on multi-resolutions texture image information (MRTII). The features inspired by human visual perception of the spectrogram image could provide significant classification for real-life emotional recognition. In addition, it could improve the accuracy among different language databases, which is more general than our work. However, our targeted work can achieve a better result in our research filed.

Zhang et al. [19] used SVM and DBN to conduct speech emotion recognition on Chinese speech database. They also extracted five kinds of speech features as the input of SVM and DBN, but the experiments were simple, and could not reflect which feature was more effective. In this work, we tried to explore more feature groups and set different weights for different features according to their contribution to emotion recognition. In addition, the novel method that combined SVM and DBN could achieve a higher accuracy than their previous work (94.6%).

Recent research [27] shows convolutional neural network (CNN) is sensitive to the sequence of images and learns a dictionary of features that are portable across languages. Additionally, combining the recurrent neural network (RNN) with deep CNN can result in a tremendous accelleration and increase in accuracy. This work also creatively applied multiple kernel learning (MKL) to CNN, and finally achieved a high accuracy of 96.55% with feature selection. Compared with their work which took original speech signals as an input, we took certain features such as MFCC and pitch as the input of SVM and DBN, and these features have been demonstrated to be helpful to distinguish emotion. Although deep CNN can extract emotional features from speech signals automatically, feature selection can improve accuracy to some extent.

## 6. Conclusions and Future Work

In this work, we explored how to accurately recognize emotion status in speech. SVM and DBN methods were compared for speech emotion recognition. DBN could extract effective features automatically to represent different emotion status. However, the parameter optimization for DBN was complex and time consuming. Therefore, we proposed a novel classification method that combines SVM and DBN as an end-to-end classifier, in which DBN acted as a deep feature extractor and SVM was a classifier. Combing them could achieve a better result than that of using only SVM or DBN. It demonstrated that deep features could reflect the high-level emotion status using low-level speech features, which was convenient as this approach could save a lot of manual work on neural network optimization. So SVM and DBN combined approach was very simple to use. Our tests showed that the proposed approach could work very well for small training samples such as the Chinese speech dataset we used.

In the future, we will test this approach with larger speech datasets. In addition, lexical features contain large amounts of information that can be potentially used [28]. If we can make use of lexical features combined with speech features, the performance of speech emotion recognition should be improved.

## Figures and Tables

**Table 1:** Attribute of Chinese speech database.

Corpus	Access	Language	Size	Subject	Emotions
CASIA	Commercially available	Chinese	400 utterances 4 actors × 6 emotions	4 professional actors	ang, fea, hap, neu, sad, sur

**Table 2:** Accuracy of male and female group using SVM.

Emotion	Male (%)	Female (%)
Angry	90	82
Fear	88	86
Happy	84	82
Neutral	80	88
Sad	86	88
Surprise	82	82
Average	85	84.6

**Table 3:** Accuracy of male and female group using DBN.

Emotion	Male (%)	Female (%)
Angry	92	94
Fear	94	96
Happy	90	96
Neutral	98	96
Sad	98	90
Surprise	96	96
Average	94.6	94.6

**Table 4:** Accuracy of male and female group using combining algorithm.

Emotion	Male (%)	Female (%)
Angry	96	98
Fear	98	96
Happy	94	96
Neutral	96	96
Sad	94	94
Surprise	98	94
Average	96	95.6

**Table 5:** Summary of related work.

Author	Database Size	Number of Emotions	Subjects	Gender-Dependent	Classifier	Features
Amiya Kumar	4200 utterances	7	5 native speaker	No	SVM	Statistic
Zhaocheng Huang	12 h	4	professional actors	No	GMM	Intrinsic
Zixing Zhang	18,216 utterances	11	51 children	No	SVM	Intrinsic
Arti Rawat	10 utterances	5	5 people	No	NN	Intrinsic
Ya Li	535 utterances	7	10 professional actors	No	SVM	Statistic
Jinkyu Lee	12 h	4	professional actors	Yes	RNN	Intrinsic
Kunching Wang	1584 utterances	4	professional actors	No	SVM	Statistic
Weishan Zhang	1200 utterances	6	professional actors	Yes	DBN	Statistic
